# Slicing and dicing ARDS: we almost forgot the lungs

**DOI:** 10.1186/s13054-021-03611-8

**Published:** 2021-05-28

**Authors:** Marry R. Smit, Lieuwe D. J. Bos

**Affiliations:** 1 Department of Intensive Care, Amsterdam UMC, location AMC, University of Amsterdam, Meibergdreef 9, 1105 AZ Amsterdam, The Netherlands; 2 Laboratory of Experimental Intensive Care and Anesthesiology, Amsterdam UMC, location AMC, University of Amsterdam, Meibergdreef 9, 1105 AZ Amsterdam, The Netherlands; 3 Department of Respiratory Medicine, Amsterdam UMC, location AMC, University of Amsterdam, Meibergdreef 9, 1105 AZ Amsterdam, The Netherlands

## Introduction

The acute respiratory distress syndrome (ARDS) is a critical illness characterized by severe lung inflammation and pulmonary oedema caused by increased alveolar permeability. Considerable etiological, physiological and biological heterogeneity is apparent in patients with ARDS, which has likely hampered clinical trials to show benefit of treatment strategies [1]. The promise of precision medicine is that outcomes can be improved through the identification of homogeneous groups (the so-called ARDS phenotypes) that do benefit from a specific treatment [2].

Calfee et al. [3] proposed ARDS phenotypes based on latent class analysis (LCA) of a combination of plasma biomarkers and clinical characteristics and found that mortality was higher in the ‘hyper-inflammatory’ than in the ‘hypo-inflammatory’ phenotype (prognostic enrichment). Importantly, systemic inflammatory phenotypes showed differential responses to positive end-expiratory pressure (PEEP) strategy, fluid strategy and administration of simvastatin. These hallmark studies have shown that ARDS can be repeatedly and reliably sliced into more homogenous portions. The main limitation of this approach is that plasma biomarkers do not necessarily reflect pulmonary disease in critically ill patients with multiple organ failure and indeed these phenotypes could also be recognized in patients without ARDS [4].

Distinct lung morphological patterns, namely focal, diffuse and patchy (the last two together are also called non-focal), have been identified in the early 2000s through physician-driven pattern recognition [5]. In the LIVE trial, patients were randomized to standard of care or personalized mechanical ventilation. Patients in the intervention arm were treated with prone positioning in case of a focal lung morphology and with recruitment manoeuvres in case of a non-focal lung morphology [6]. This study failed to show benefit of personalized ventilation in the intention-to-treat analysis, as it was hampered by a large proportion of misclassifications. Correctly classified patients did seem to benefit from the personalized intervention, while misclassified patients had a high mortality rate. Misclassifications are driven by the lack of an algorithmic approach to morphology assessment.

Recently in this journal, Wendel Garcia et al. [7] proposed new phenotypes for ARDS based on respiratory mechanics, gas-exchange and computed tomography (CT)-derived measurements of lung tissue. They piled data from 238 patients originating from multiple studies where CT-scans, respiratory mechanics and blood gas analyses were systematically collected. LCA based on data collected at PEEP 5 cmH_2_O revealed two distinct phenotypes. About half the patients showed larger amount of dead space, more non-inflated lung tissue and lower PaO_2_/FiO_2_ ratio. This group was termed the ‘recruitable’ phenotype because after recruitment and increase of PEEP to 15 cmH_2_O they showed improved gas-exchange and lung aeration (predictive enrichment). The other patients had a larger proportion of well-aerated lung tissue, a higher PaO_2_/FiO_2_ ratio and less dead space were classified as the ‘non-recruitable’ phenotype as they did not show gas-exchange or re-aeration benefit from the recruitment manoeuvre. The authors found that ICU mortality was higher in the ‘recruitable’ phenotype compared to the ‘non-recruitable’ phenotype.

The evident novelty of the study is the use of LCA to quantify differences between phenotypes that are traditionally in the eye of the beholder. The analysis confirms our clinical suspicion that there are distinct subgroups of ARDS patients and that some might benefit from recruitment while others will not. Yet, the relative difficulty of using CT analysis to phenotype these patients is evident given the need for 16 years of recruitment to include 238 patients with ARDS. Furthermore, these CT’s need to be segmented, which requires timely, manual labour and patients need to be transported to the radiology department at 5 cmH_2_O PEEP with a severity of hypoxemia that would not be acceptable for transport for many physicians. Indeed, this limitation was reflected in the LIVE trial where chest CT-scans at PEEP 5 cmH_2_O were commonly not feasible because of the risk of transportation [6].

**Fig. 1:**
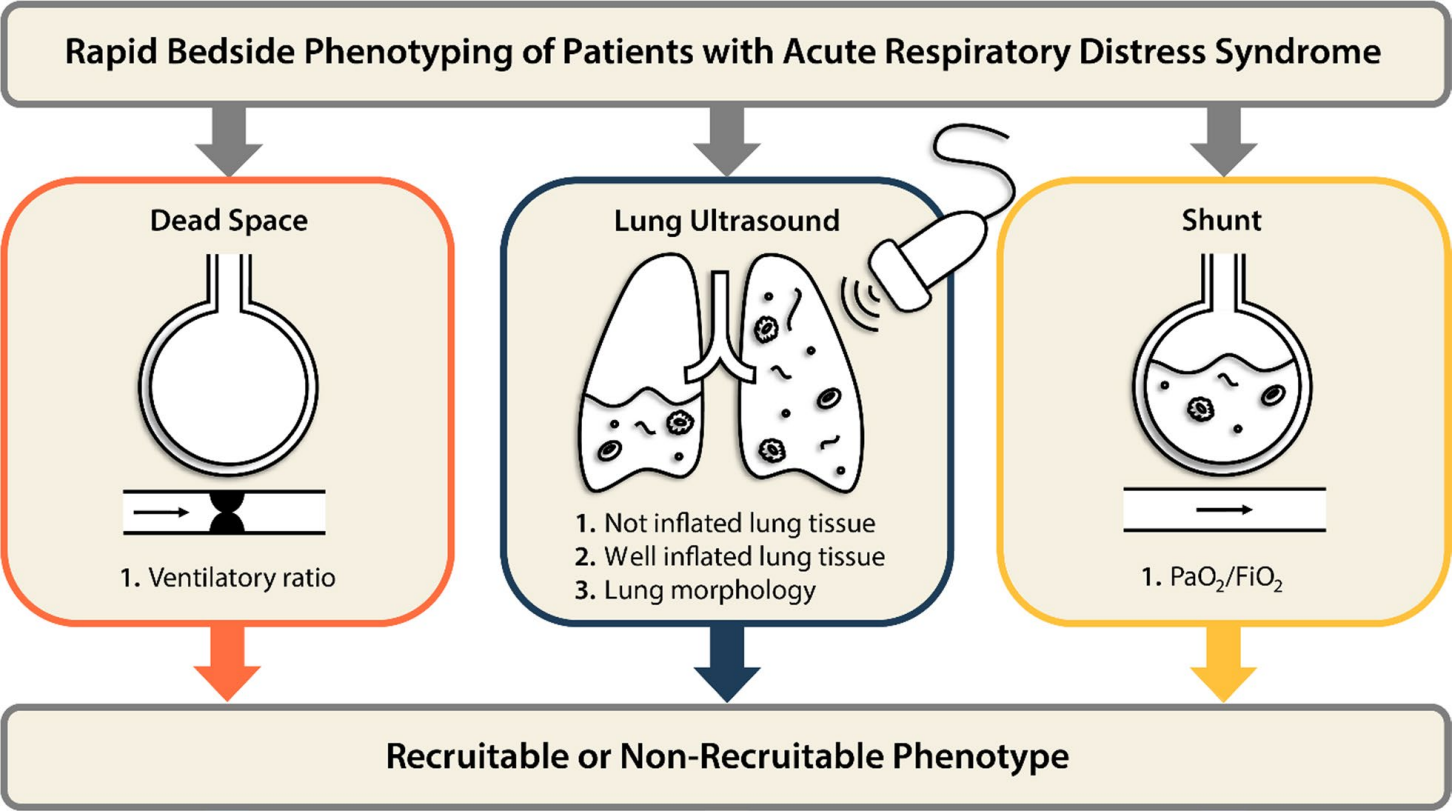
These proposed methods could potentially facilitate bedside phenotyping of patients with ARDS and are widely available across ICU’s. Phenotyping of ARDS patients should ideally be performed within 24 h after diagnosis, whereafter ventilator management of the patient can be modified based on the classified phenotype

Phenotype approaches for ARDS patients should be designed in a way that they can be implemented in ICU of all sorts and sizes [8]. And while the PaO_2_/FiO_2_ ratio as an indirect measure of shunt is widely available across ICU’s, CT-scans or volumetric capnography are not. Lung ultrasound (LUS) could play an important role in ARDS phenotypes that involve imaging parameters. LUS knows many advantages as it is fast to perform, radiation free and thus can be repeated as often as needed. Moreover, LUS avoids the need for risky transportation to the radiology department and is available in nearly every hospital [9]. Recently, a study performed by Costamagna et al. [10] showed that LUS aeration scores from the easy accessible anterior regions of a 12-region exam could accurately classify lung morphology in ARDS patients. Although this study was hampered by the single-centre setting and the very low number of patients with focal ARDS, it clearly shows the potential for LUS in assessment of lung morphology. Importantly, previous studies showed that the amount of non-aerated and well-aerated lung tissue, which was the most important CT-derived parameter separating the phenotypes, can be accurately estimated with LUS [11]. Combining LUS with indirect measurements of shunt and dead space may further improve clinical applicability and large-scale validation of the pulmonary phenotyping (Fig. 1). For example, the ventilatory ratio might be a good surrogate for alveolar dead space and can be calculated at the bedside without the scarcely available volumetric capnography [12].

In conclusion, the heterogeneous syndrome of ARDS was sliced into phenotypes by markers of systemic inflammation and is now further diced by a combination of parameters of gas-exchange abnormality and CT-estimated lung weight. Just like we can’t expect every family to slice and dice like a top chef and serve a Michelin star dinner, the evident clinical challenge lies in making cuts of ARDS heterogeneity widely available. The way forward is widespread collaboration between researchers and clinicians, using commonly available bedside measurements in large patient populations to further evaluate the clinical applicability of the proposed phenotyping schemes.

## Data Availability

Not applicable.
